# Whole genome sequencing of a novel sea anemone (*Actinostola* sp.) from a deep-sea hydrothermal vent

**DOI:** 10.1038/s41597-024-02944-7

**Published:** 2024-01-22

**Authors:** Chang Liu, Chao Bian, Qiang Gao, Zijian Gao, Yu Huang, Lingling Wang, Qiong Shi, Linsheng Song

**Affiliations:** 1 Liaoning Key Laboratory of Marine Animal Immunology, Dalian Ocean University, Dalian, 116023 China; 2 Laboratory of Aquatic Genomics, College of Life Sciences and Oceanography, Shenzhen University, Shenzhen, 518057 China; 3 Southern Laboratory of Ocean Science and Engineering, Zhuhai, 519000 China; 4 Shenzhen Key Lab of Marine Genomics, BGI Academy of Marine Sciences, BGI Marine, Shenzhen, 518081 China

**Keywords:** Behavioural genetics, Evolutionary genetics

## Abstract

Deep-sea hydrothermal vents are usually considered as extreme environments with high pressure, high temperature, scarce food, and chemical toxicity, while many local inhabitants have evolved special adaptive mechanisms for residence in this representative ecosystem. In this study, we constructed a high-quality genome assembly for a novel deep-sea anemone species (*Actinostola* sp.) that was resident at a depth of 2,971 m in an Edmond vent along the central Indian Ocean ridge, with a total size of 424.3 Mb and a scaffold N50 of 383 kb. The assembled genome contained 265 Mb of repetitive sequences and 20,812 protein-coding genes. Taken together, our reference genome provides a valuable genetic resource for exploring the evolution and adaptive clues of this deep-sea anemone.

## Background & Summary

Deep-sea hydrothermal vents are a representative ecosystem, where hot and chemical fluids exit the seafloor from black smoker chimneys^1^. These vents are considered as extremely harsh environments with high pressure, high temperature, low oxygen, and high concentrations of methane (CH_4_), heavy metals and hydrogen sulfide (H_2_S)^2,3^. Many species live within and around these hydrothermal vents, including various crabs, shrimps, fishes, octopus, as well as diverse sessile creatures such as sea anemones, barnacles, and tube worms^4,5^. These special organisms arouse many interests to developers for drugs, enzymes, cosmetics, biofuel, and other products. However, the genetic basis of evolution and adaptation of deep-sea hydrothermal vents animals is still lacking.

**Fig. 1:**
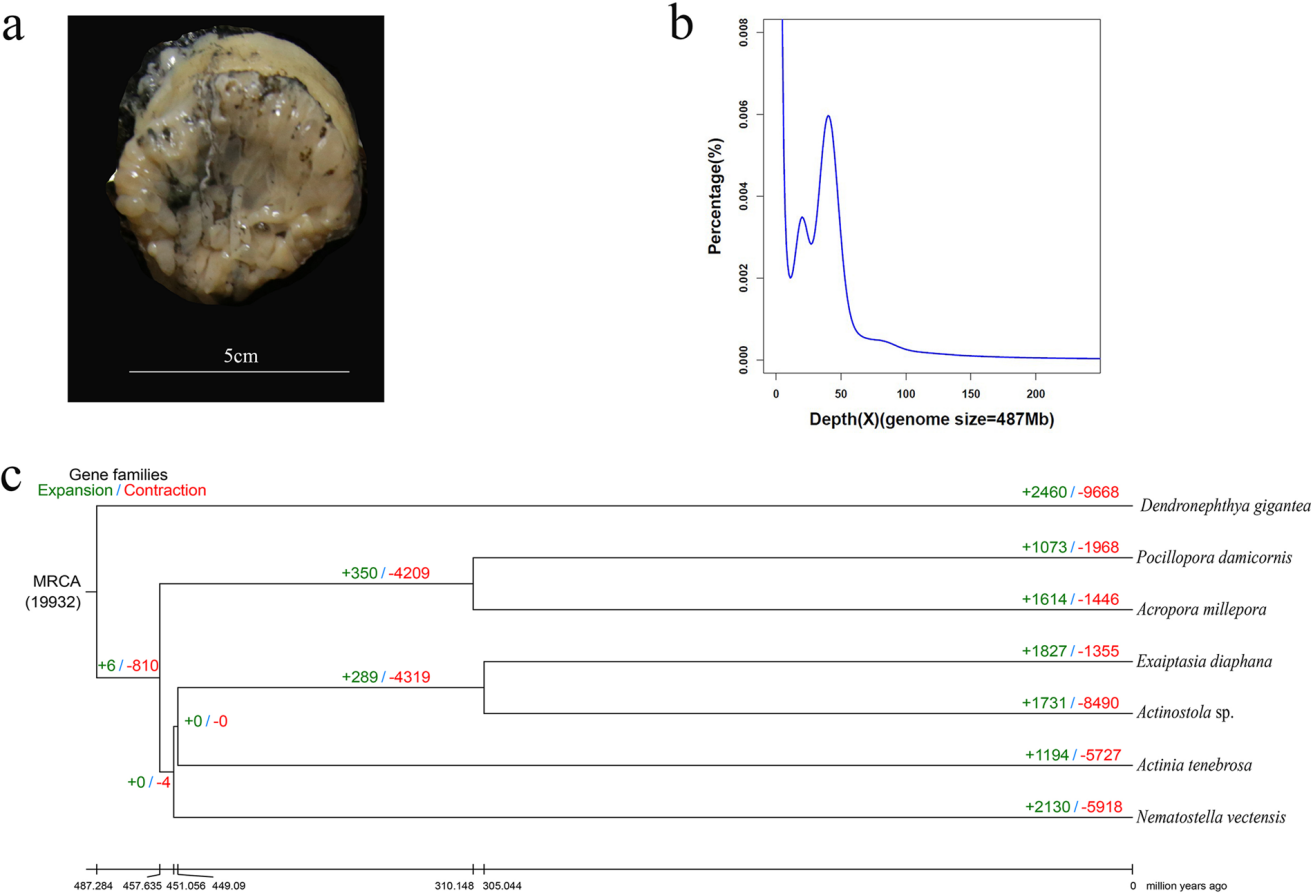
Sampling details and comparative analyses of the deep-sea anemone. (**a**) Image of the sequenced *Actinostola* sp. (**b**) Genome survey. (**c**) Gene family analysis and divergence time of seven representative Cnidaria species.

Sea anemones, a group of primitive Cnidarians, are widely distributed across the whole ocean depth^6^. Their unique adaptive strategies help them live in a variety of marine habitats from shallow waters to deep-sea trenches. During a recent expedition, an anemone (Fig. 1a) was collected at 2,971 m depth in certain hydrothermal vents of Indian Ocean (E60.5, N6.4). In this area, Actinostolidae anemones showed the highest abundance reported from previous research^7^. Morphological and molecular analyses suggest that this deep-sea anemone belongs to the genus *Actinostola*. Here, whole genome sequencing was performed to construct a high-quality genome assembly for this newfound *Actinostola* sp., which will help to elucidate adaptive clues to deep-sea hydrothermal environments.

A total of 44.23-Gb paired-end reads produced by an Illumina sequencing platform were used for a genome survey (Fig. 1b). The sequencing depth with the highest frequency was identified at 54, and the total number of 17-mer reads was 19,503,242,454. Therefore, the estimated genome size of *Actinostola* sp. was about 487 Mb. Meanwhile, the heterozygosity rate of this genome was predicted to be 0.9% (see more details in Fig. 1b).

A 424.3-Mb draft genome was subsequently assembled based on 112.37-Gb long reads generated from a PacBio sequencing platform and 26.10-Gb short reads generated from an Illumina Hiseq Xten platform, with a contig N50 of 373 kb, a scaffold N50 of 383 kb and GC content of 38.7% (Table 1). The routine BUSCO (Benchmarking Universal Single-Copy Orthologs) method was applied to evaluate the completeness of our assembled genome, using the eukaryota_odb9 database as the reference. Finally, 252 (83.2%) BUSCO core genes were completely identified.

**Table 1:** Summary of the genome assembly for the sequenced *Actinostola* sp.

Genome assembly	Data
Contig N50 (kb)	372.9
Contig number (>100 bp)	1779
Scaffold N50 (kb)	383.1
Scaffold number (>100 bp)	1596
Total length (Mb)	424.3
Genome coverage (×)	280
**Genome annotation**	**Data**
Protein-coding gene number	20,812
Mean transcript length (bp)	9,353
Mean exons per gene	6.31
Mean exon length (bp)	240.89
Mean intron length (bp)	1,442

For further repeat annotation, a total of 265-Mb data covering 62.4% of the total assembled genome were predicted to be repeat sequences. Among them, 25.5% of the genome (108.2 Mb) was DNA repeat elements, 8.4% (35.6 Mb) was long interspersed nuclear elements (LINE), 14.3% (60.6 Mb) was long terminal repeats (LTR), and 0.8% (3.6 Mb) was short interspersed nuclear elements (SINE). After masking those repetitive regions, we applied an integrated method of homologous sequence search and *de novo* gene prediction to obtain annotations of 20,812 protein-coding genes in the assembled genome. By searching four public databases including GO (Gene ontology)^8^, KEGG (Kyoto Encyclopedia of Genes and Genomes)^9^, SwissProt^10^ and TrEMBL^11^, we found that 97.89% (19,111 in total) of these predicted genes were functionally annotated.

The coding sequences (CDS), predicted from assembled genomes of *Actinostola* sp. (this study) and other seven representative species (Fig. 1c), were utilized for clustering of gene families. Eventually, the 20,812 protein-coding genes of *Actinostola* sp. were clustered into 10,327 gene families, among them 3,526 were single-copy orthologous. A phylogenetic tree (Fig. 1c) was constructed based on these single-copy orthologous gene families with the maximum likelihood method, predicting that the divergence of our newfound *Actinostola* sp. from another sea anemone *Exaiotasia diaphana* occurred 305 million years ago (Mya). This high-quality reference genome for *Actinostola* sp. can also provide novel insights for enhancing wild resource conservation, discovering new functional genes, developing novel marine drugs, and elucidating special adaptive mechanisms.

## Methods

### Sample collection, library construction, and genome sequencing

A specimen of the *Actinostola* sp. was collected from an Edmond vent along the central Indian Ocean ridge for whole genome sequencing. Genomic DNA (gDNA) was extracted using QIAwave DNA Blood & Tissue Kit (Qiagen, Germantown, MD, USA). The genome was sequenced using a combination of sequencing techniques, including paired-end sequencing with a 500-bp inserted library on an Illumina Hiseq Xten platform (Illumina Inc., San Diego, CA, USA), and a PacBio library with an insert-size of 20 kb on a PacBio sequencing platform (Pacific Biosciences, Menlo Park, CA, USA).

### Genome size estimation

The Illumina short reads were filtered with SOAPfilter v2.2^12^. Clean reads were then used for estimation of the *Actinostola* sp. genome size with a 17-mer frequency distribution analysis according to the following formula^13^: Genome Size = Kmer_num/peak_depth, where k-mer_num is the total number of reads and peak_depth denotes the estimated peak frequency of 17-mers.

### Genome assembly

Before assembly, the PacBio long sequencing reads were calibrated using LoRDEC^14^, along with the clean Illumina short reads. After correction, DBG2OLC^15^ was applied to assemble these long reads to contigs with assistance of the clean short reads. To further improve the genome accuracy, two rounds of polishing was performed with different strategies. First, Racon v1.3.1^16^ was employed for contigs polishing based on the uncorrected PacBio long reads. Second, the clean short reads were used to polish the contigs with pilon^17^. After heterozygosity reducing with Redundans^18^, we obtained a polished genome assembly for the sequenced *Actinostola* sp. BUSCO^19^ v5.22 provided quantitative measurements for the completeness of this assembly with the popular eukaryota_odb9 database as the reference.

### Genome annotation

We predicted repeat elements by *de novo* and homology annotations. RepeatModeler^20^ and LTR-FINDER^21^ were employed for the *de novo* prediction to build a repeat library. Then, the two libraries were combined and aligned to the assembled genome with RepeatMasker^22^. For the homology prediction, a known repeat library (Repbase^23^) was employed to identify repeats with RepeatMasker and RepeatProteinMask^22^. Tandem repeats were detected using Tandem Repeat Finder^24^. Finally, by integrating these data from both methods, a nonredundant set of repeat elements were obtained.

To predict protein-coding genes, protein sequences form nine representative species including California sea hare (*Aplysia californica*), nematode (*Caenorhabditis elegans*), sacoglossan sea slug (*Elysia chlorotica*), limpet (*Lottia gigantea*), two-spot octopus (*Octopus bimaculoides*), invasive apple snail (*Pomacea canaliculata*), glass anemone (*Exaiptasia pallida*), starlet sea anemone (*Nematostella vectensis*), and human (*Homo sapiens*), were downloaded from Ensembl^25^, and then they were mapped to our assembled genome with TBLASTn^26^. Subsequently, gene structures were predicted by GeneWise^27^. Finally, we integrated all these predicted results using MAKER^28^ to obtain a consistent gene set.

For functional annotation, BLASTp^29^ was applied to align the predicted protein sequences against four public databases (including SwissProt^10^, TrEMBL^10^, KEGG^30^ and InterPro^8^), and then these results were retrieved to obtain GO^31^ terms.

## Data Records

Our final assembly and annotation data have been deposited at the NCBI with accession number JAUJYZ000000000^32^. Protein and gene coding sequences are uploaded into FigShare depository for public accession^33^. The raw reads of PacBio and Illumina sequencing were also uploaded at the NCBI with accession numbers SRR25988563- SRR25988567^34^.

## Technical Validation

The genome assembly was 424.3 Mb with a scaffold N50 of 383 kb. For quantitative assessment of this genome assembly, we showed that 83.2% of the reference BUSCO genes (insecta_db9) were successfully identified in the final genome assembly version, suggesting remarkable completeness of this *Actinostola* sp. genome assembly.

## Data Availability

No custom scripts or code was used in this study. All software and pipelines were executed according to the manuals and protocols of related published bioinformatic tools. Corresponding versions and codes/parameters of software have been described in Methods.

## References

[1] Van Dover CL, Trask JL (2000). Diversity at deep-sea hydrothermal vent and intertidal mussel beds. Marine Ecology Progress Series.

[2] Little CTS, Vrijenhoek RC (2003). Are hydrothermal vent animals living fossils?. Trends in Ecology & Evolution.

[3] Sun SE, Sha Z, Xiao N (2021). The first two complete mitogenomes of the order Apodida from deep-sea chemoautotrophic environments: New insights into the gene rearrangement, origin and evolution of the deep-sea sea cucumbers. Comparative Biochemistry and Physiology Part D: Genomics and Proteomics.

[4] Tunnicliffe, V., McArthur, A. G. & McHugh, D. in *Advances in marine biology* Vol. 34 353–442 (Elsevier, 1998).

[5] Zierenberg RA, Adams MWW, Arp AJ (2000). Life in extreme environments: Hydrothermal vents. Proceedings of the National Academy of Sciences.

[6] Jamieson, A. *The hadal zone: life in the deepest oceans*. (Cambridge University Press, 2015).

[7] Zhou Y (2018). Characterization of vent fauna at three hydrothermal vent fields on the Southwest Indian Ridge: Implications for biogeography and interannual dynamics on ultraslow-spreading ridges. Deep Sea Research Part I Oceanographic Research Papers.

[8] Hunter S (2009). InterPro: the integrative protein signature database. Nucleic acids research.

[9] Ogata H (1999). KEGG: Kyoto encyclopedia of genes and genomes. Nucleic acids research.

[10] Boeckmann B (2003). The SWISS-PROT protein knowledgebase and its supplement TrEMBL in 2003. Nucleic acids research.

[11] Kulikova T (2004). The EMBL nucleotide sequence database. Nucleic Acids Research.

[12] Chen Y (2018). SOAPnuke: a MapReduce acceleration-supported software for integrated quality control and preprocessing of high-throughput sequencing data. Gigascience.

[13] Hequan S, Jia D, Mathieu P, Korbinian S (2018). findGSE: estimating genome size variation within human and Arabidopsis using k-mer frequencies. Bioinformatics.

[14] Salmela L, Rivals E (2014). LoRDEC: accurate and efficient long read error correction. Bioinformatics.

[15] Ye C, Hill CM, Wu S, Ruan J, Ma Z (2016). DBG2OLC: efficient assembly of large genomes using long erroneous reads of the third generation sequencing technologies. Scientific reports.

[16] Vaser R, Sović I, Nagarajan N, Šikić M (2017). Fast and accurate de novo genome assembly from long uncorrected reads. Genome research.

[17] Walker BJ (2014). Pilon: an integrated tool for comprehensive microbial variant detection and genome assembly improvement. PloS one.

[18] Pryszcz LP, Gabaldón T (2016). Redundans: an assembly pipeline for highly heterozygous genomes. Nucleic acids research.

[19] Simão FA, Waterhouse RM, Ioannidis P, Kriventseva EV, Zdobnov EM (2015). BUSCO: assessing genome assembly and annotation completeness with single-copy orthologs. Bioinformatics.

[20] Smit, A., Hubley, R. & Green, P. RepeatModeler Open-1.0. 2008–2010. *Access date Dec* (2014).

[21] Xu Z, Wang H (2007). LTR_FINDER: an efficient tool for the prediction of full-length LTR retrotransposons. Nucleic acids research.

[22] Chen N (2004). Using Repeat Masker to identify repetitive elements in genomic sequences. Current protocols in bioinformatics.

[23] Jurka J (2005). Repbase Update, a database of eukaryotic repetitive elements. Cytogenetic genome research.

[24] Benson G (1999). Tandem repeats finder: a program to analyze DNA sequences. Nucleic acids research.

[25] Flicek P (2012). Ensembl 2013. Nucleic acids research.

[26] Gertz EM, Yu Y-K, Agarwala R, Schäffer AA, Altschul SF (2006). Composition-based statistics and translated nucleotide searches: improving the TBLASTN module of BLAST. BMC biology.

[27] Birney E, Clamp M, Durbin R (2004). GeneWise and genomewise. Genome research.

[28] Cantarel BL (2008). MAKER: an easy-to-use annotation pipeline designed for emerging model organism genomes. Genome research.

[29] Altschul SF, Gish W, Miller W, Myers EW, Lipman DJ (1990). Basic local alignment search tool. Journal of molecular biology.

[30] Kanehisa M, Goto S (2000). KEGG: kyoto encyclopedia of genes and genomes. Nucleic acids research.

[31] Consortium GO (2004). The Gene Ontology (GO) database and informatics resource. Nucleic acids research.

[32] Bian C (2023). NCBI GenBank.

[33] Bian C (2023). figshare.

[34] (2023). NCBI Sequence Read Archive.

